# Improvement of gamete quality by stimulating and feeding the endogenous antioxidant system: mechanisms, clinical results, insights on gene-environment interactions and the role of diet

**DOI:** 10.1007/s10815-016-0767-4

**Published:** 2016-07-16

**Authors:** Maurizio Dattilo, D’Amato Giuseppe, Caroppo Ettore, Yves Ménézo

**Affiliations:** 1 Parthenogen, Via F. Pelli 1, Lugano, 6900 Switzerland; 2 ASL Bari, U.O. Fisiopatologia della Riproduzione Umana e PMA, Conversano, Ba Italy; 3 London Fertility Associates, 104 Harley Street, London, UK

**Keywords:** Sperm, Oocyte, Oxidative stress, DNA methylation, Epigenesis

## Abstract

Oxidative damage triggers extensive repair in gametes and thereafter in the zygote but it results in clinically relevant damage when affecting the maturation of the gametes chromatin, i.e. padlocking and epigenetic marking. It associates with defective DNA methylation and/or with oxidation of the methyl marks leading to derangement of gamete epigenetics, defects of chromatin condensation and aneuploidy. A proper feed to the one carbon cycle has the potential to stimulate the endogenous antioxidant defences, i.e. gluthatione synthesis, and to activate compensative homeostatic mechanisms restoring both the oxy-redox balance and DNA methylation, which are indeed strictly cross-regulated. This has been shown to produce measurable clinical improvements of male reproductive potential in pilot studies herein summarised. However, the effects of dietary habits and of supplementations are variable according to the individual genetic substrate, as genetic variants of several of the concerned enzymes occur with high frequency. Individual risk assessments and personalised interventions are still difficult to implement, in the meantime, a very varied diet may facilitate metabolic compensation in the majority of the cases. This review aims to report on the mechanisms of damage, on the opportunities to modulate the physiologic oxy-redox homeostasis by means of a varied diet or dietary supplements and on the open issues related to the genetic variability of the population.

## Introduction

The energy supply to life on the Earth planet is mostly based on oxygen metabolism, and any healthy cell is well equipped to manage the possible damage from oxidising agents. Damage will occur only when the load of oxidative species exceeds the capacity of the cellular antioxidant mechanisms, which are largely dependent on the assumption of dietary substrates and micronutrients [1] and on the individual genetic substrate [2].

Among the cellular species, gametes are the most sensitive to these imbalances due to their long maturation process in contact with the environment, which is particularly true for the male gametes. Evolution selected this type of process likely because gametes have the specific task to collect environment signals and to pass them to the offspring in the form of epigenetic marks [3]. This leads to two simple concepts: First, only a well-balanced metabolism will support proper gamete maturation and their reproductive competence; Secondly, as gamete deterioration is sensitive to environmental signals, similarly their improvements should be sensitive to feeding signals, if we know which the good ones are.

Therefore, it is worth putting more effort into the comprehension of oxy-redox homeostasis, the consequences of supporting interventions and the complex interaction with the environment, including feeding habits and genetic variants in the population.

### Oxidative damage affects gamete chromatin and epigenetics

Oxidative molecular damage occurs in all cells but it is very likely to be repaired. Also spermatids, oocytes and thereafter zygotes possess powerful, although finite, DNA repair capacity [4]. On the other side, compensative or repairing mechanisms capable of correcting damage beyond simple molecular oxidations, i.e. to the chromatin structure, have not yet been described. It is now evident that, besides the genes, the chromosomes of gametes provide a structural framework that is required for proper embryonic development [5]. Oxidative aggression may affect the chromatin structure and the epigenetic regulation of gametes in at least two relevant manners: in sperm, by affecting the protamin content [6]; In both sperm cells and oocytes by misleading the epigenetic marks, i.e. by affecting DNA methylation patterns.

The methylation of DNA occurs at cytosine residues, parts of “cytosine-phosphate binding-guanosine” repeats called CpGs. The promoters of many genes contain areas enriched of CpG repeats called CpG islands. The extensive methylation of the CpG islands causes loss of affinity of the gene promoters for the transcription factors and results in gene silencing [7]. In addition, the methylation of CpGs, together with histone lysine methylation [8], also regulates chromatin state so that extensive methylation is followed by chromatin compaction to generate a transcriptionally silent state that is an essential maturation phase of both sperm [9] and oocytes [10].

**Fig. 1:**
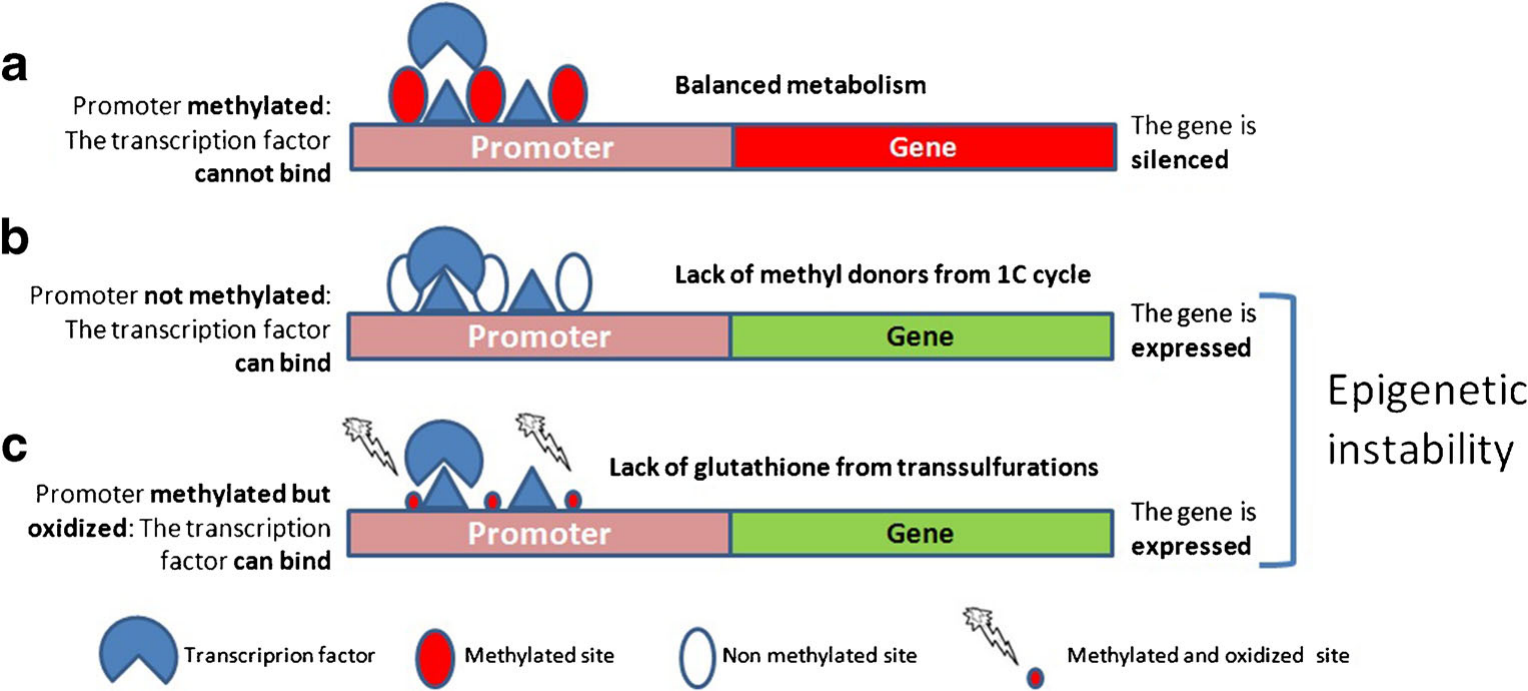
Epigenetic instability from oxidative damage. **a** The methylation of cytosine (*filled circles*) within CpG repeats to form methyl cytosine causes loss of affinity of the transcription factors for the promoter and silencing of the gene. **b** If the cytosines are not methylated (*empty circles*) due to lack of SAMe or if the methylation mark is lost due to de-methylation triggered by methyl cytosine oxidation, the gene is not anymore silenced and may undergo inappropriate expression. **c** If either the methyl cytosine or the guanosine of the CpG repeat are oxidised (*small filled circles*), the domain reverts from hydrophobic back to hydrophilic and the transcription factors recover the ability to bind and to express the gene in spite of the methylation of the CpG island. Both cases **b** and **c** lead to epigenetic instability

The CpGs and the CpG islands are the main target of oxidative damage. The oxidation of the methylated cytosines within CpGs produces Hydroxy-methyl-Cytosine (OH-mC), which is the starting step for active demethylation [11] and may result in loss of epigenetic marks. In addition, the oxidation of either methyl-cytosine or guanosine (to form 8-Oxo-Guanosine) within otherwise properly methylated CpG islands causes loss of the inhibition of the binding of transcription factors, i.e. loss of the epigenetic regulation (Fig. 1). This has been proposed as a main mechanism for the loss of epigenetic regulation following environmental damage in neoplastic [12] and neurodegenerative diseases [13] and is likely to be the most clinically relevant consequence of excessive oxidative exposure: The loss of epigenetic regulation in gametes leads to the risk of an erratic phenotype, which will certainly affect the fertilization potential as well as the activation, viability and the development of the generated embryos.

Oxidative stress-induced perturbations of DNA methylation also affects chromosome stability and segregation and may lead to aneuploidy of both sperm and oocytes. Meiosis requires full chromatin condensation to facilitate the process [14], which is called heterochromatin state. Only the areas intended for homologous chromosome recombination, the recombination hotspots, remain hypomethylated and decondensed to allow the recombination process [15]. Thus, hypomethylation of CpG islands attracts the recombination machinery, which has been well shown in germline dog models [16]. In mice oocytes, the abnormal axial chromatid condensation during meiosis alters chromosome segregation by interfering with both chromosome-microtubule interactions and sister chromatid separation [17]. Similar mechanisms seem to be in place in spermatogenesis and to have an impact on male fertility [18]. The oxidation of chromosome telomeres also contributes to gamete aneuploidy. Telomeres consist of guanosine enriched, repeated TTAGGG sequences that are several thousand base pairs long. Telomere GGG repeats where shown to be a preferential target of oxidative damage in human primary fibroblasts: while genomic damage gets extensively repaired, telomere GGG oxidation persists and leads to telomere shortening and chromosomal instability [19]. Differently from fibroblasts, gametes express telomerase activity and may repair the damage; nevertheless, human oocytes with telomere DNA deficiency are prone to aneuploidy development during meiosis [20]. Furthermore, oxidative damage causes overriding of the spindle check point, the safeguard mechanism that halts anaphase onset until meiotic spindle assembly, which provides a further link between oocyte aneuploidy and oxidative stress [21].

In summary, oxidative damage is likely to be of clinical relevance when and if resulting in derangement of epigenetic regulation as well as in perturbation of the gamete chromatin condensation phase, which does not leverage on repair mechanisms. The supportive interventions should be aimed at restoring both the full efficiency of the 1 carbon cycle (1CC) pathway, ensuring timed availability of activated methyl groups for DNA methylation, and proper reactivity by the intracellular antioxidant system, that will protect the epigenetic marks from misleading oxidation. Noteworthy, from a metabolic point of view, the 1CC and the antioxidant defences are a unique functional unit, i.e. two sides of the same coin [22].

### Physiologic oxy-redox homeostasis

In physiologic conditions, in both somatic and germline cells, any excess of reactive oxygen species (ROS) activates removal by means of a variety of biochemical reactions and enzymes forming an anti-oxidant cascade that leverages on a wide list of mediators. These include enzymatic (superoxide dismutase—SOD, glutathione peroxidase—GPx, catalase—CAT) and non-enzymatic (ascorbic acid—vitamin C, tocopherol—vitamin E, carotenoids, flavonoids and others) antioxidants [23]. On top of this cascade, there is a kind of redox buffering system based on a thiol antioxidant, the tripeptide glutathione (GSH) [24]. Besides acting as a cofactor for GPx, GSH can directly scavenge hydroxyl radicals and singlet oxygen. More importantly, GSH is able to regenerate the most important cellular antioxidants including vitamins C and E.

**Fig. 2:**
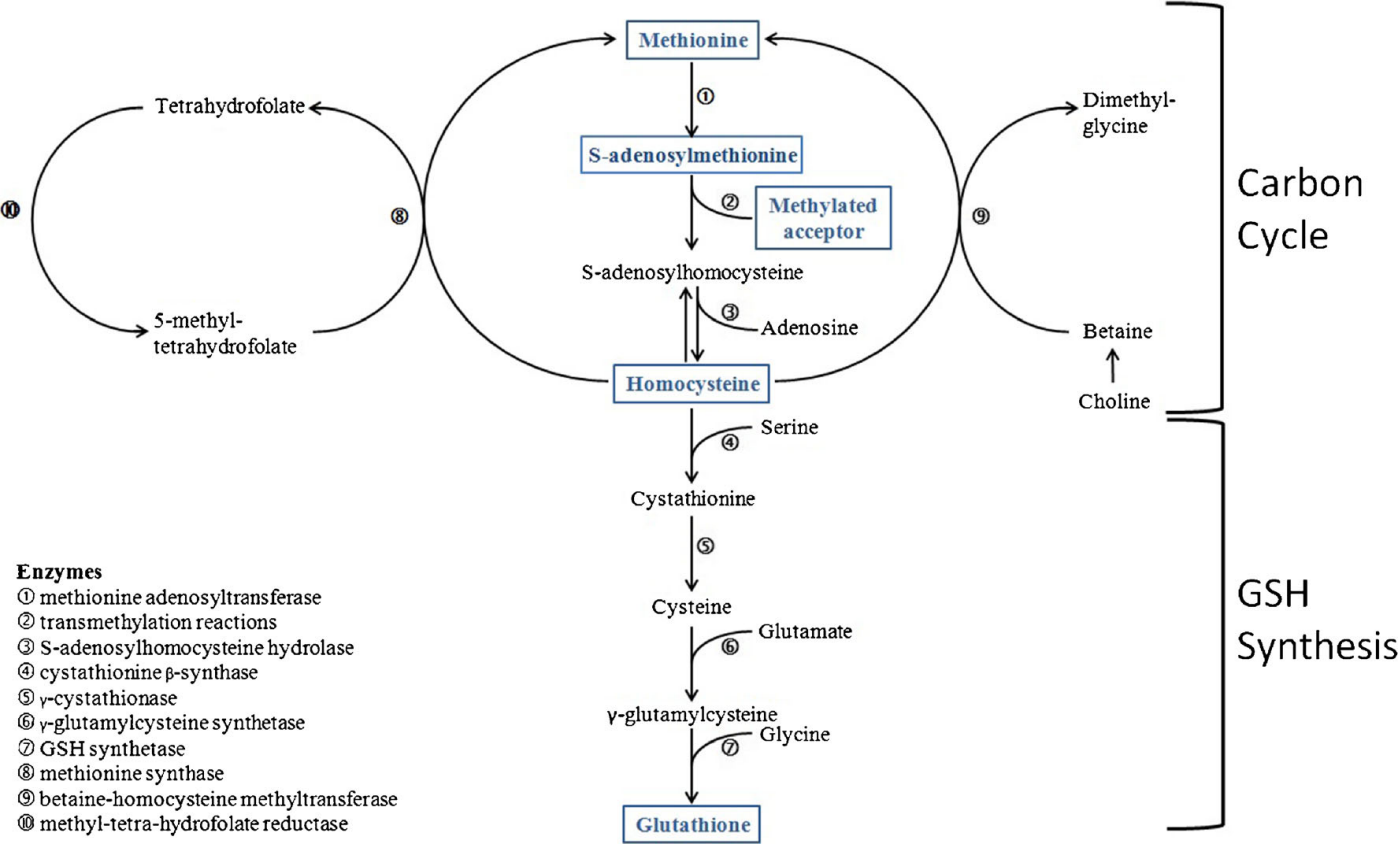
Connection between the one carbon cycle and the transsulfuration pathway (GSH synthesis). *Upper panel* The methyl group of methionine is activated by adenylation to form S-Adenosyl-Methionine (SAMe) that acts as the universal methyl donor for any acceptor including DNA: a molecule of homocysteine is formed. Homocysteine can be either re-methylated from folates or betaine or enter the transsulfuration pathway for the synthesis of glutathione (GSH). *Lower panel* Homocysteine is complexed with serine to form cystathionine and to feed the de-novo synthesis of GSH

GSH is abundant in the cytosol, the mitochondria and the nuclei of all cells [25]. It is able to release a reducing equivalent (E°) to any acceptor generating glutathione disulphide (GSSG) and can be regenerated from NADH and NDAPH. More commonly, GSH is available by de novo biosynthesis mainly by means of the so-called transsulfuration pathway, which is ubiquitous and massively cycling in all cells [26]. The starting substrate for GSH de novo biosynthesis is another sulphur but nonproteogenic aminoacid, homocysteine (Hcy), that reacts with serine to form cystathionine and thereafter binds glutamate and glycine to form the active tripeptide (Fig. 2, lower panel). The pathway for GSH synthesis is of paramount importance because GSH must be available on site to neutralise ROS just in time. There are no dietary sources or storage; The oxy-redox homeostasis is based on GSH availability by regeneration and de-novo synthesis where, when and how long it is needed within a delicate and perfect homeostatic equilibrium.

The starting substrate for GSH de novo biosynthesis is Hcy, which is in turn the end-product of the 1CC (Fig. 2, upper panel): This pathway is the base of life on the Earth planet, i.e. it is a main pathway for non-photosynthetic organisms to add the carbon unit to molecules and its massive cycling is essential for cell growth and differentiation. Briefly, the methyl group of methionine, after an activating adenylation to form S-adenosyl-methionine (SAMe), is released to any acceptor and generates Hcy. Hcy can thereafter be re-methylated to methionine with a methyl group donated by either methyl-tetrahydro-folate or betaine (trimethyl-glycine). Lack of Hcy recycling and its accumulation is highly toxic to cells, since it is a powerful pro-oxidant and a strong inhibitor of transmethylation [27].

**Fig. 3:**
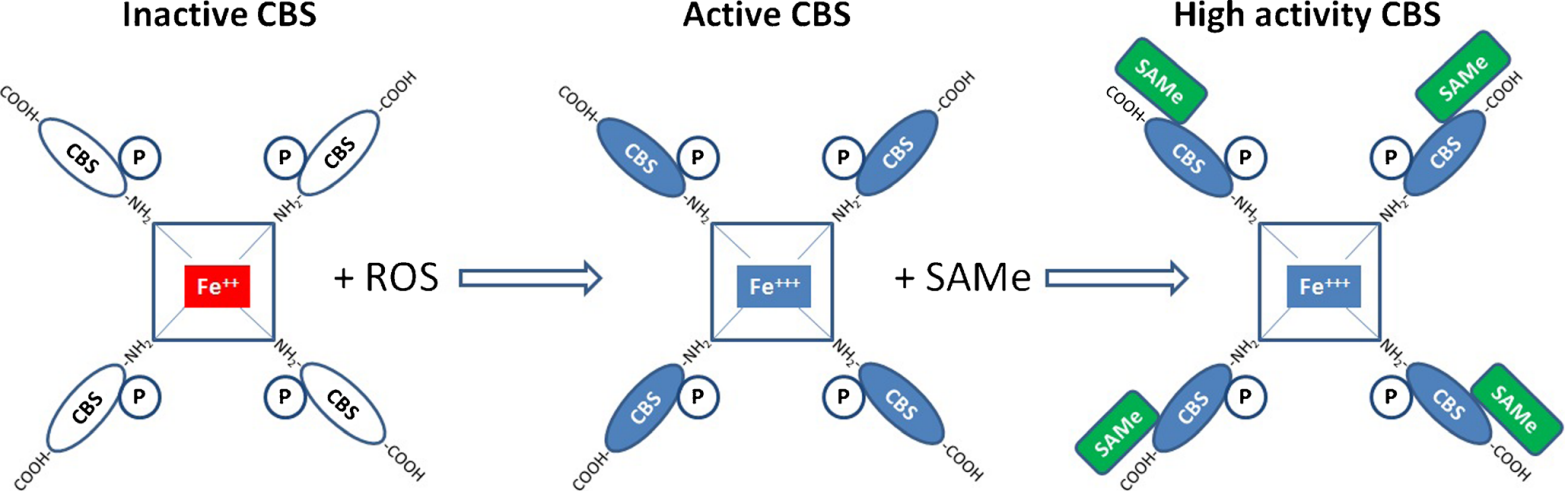
Regulation of cystathionine-beta-synthase (CBS) activity. The enzyme is formed by a tetramer of CBS domains complexed with a heme functioning as a redox sensor. The oxidation of the heme group activates the enzyme. However, a significant activity funneling homocysteine into the trannssulfuration pathway will only occur if SAMe is available and binding to the carboxy-terminal end of the CBS domains. Thus, full efficiency of the transsulfuration pathways for GSH synthesis will only occur after the requirement for activated methyl groups (SAMe) for the one carbon cycle is satisfied

Whether or not Hcy is focussed on GSH synthesis depends on the activity of a key enzyme, cystathionine beta synthase (CBS), a tetrameric enzyme having pyridoxine (vit. B6) as the essential co-factor and binding Hcy by means of zinc fingers [28]. It is highly active in the liver, pancreas and cerebral cortex; however, the gene expression has been also shown in murine testicular tissue [29] and granulosa cells where gene silencing causes follicular arrest [30]. CBS responds to a very peculiar mechanism of activation that renders this enzyme the epicentre of cellular metabolism. Briefly, the enzyme is made of 4 CBS protein domains complexed around a heme group similar to the one occurring in haemoglobin. The iron of the heme group acts as a redox sensor: In the inactive state it occurs as ferrous iron (Fe++) whereas anytime the cell environment becomes oxidative it turns in the ferric state (Fe+++). As the iron oxidises CBS enters in its active conformation configuring a typical redox regulation that opens or closes the transulfuration pathway as a reversible switch [24, 31]. However, in order to acquire high cyclicity and to generate high rate GSH output, mammalian CBS requires a second activation: a moiety of SAMe, i.e. the activated methyl group for transmethylation generated within the 1CC, must bind each CBS domain exerting an allosteric up-regulation that increases the enzyme activity approximately fivefold [28, 32] (Fig. 3).

The regulation of CBS activity, besides being the core of cell resistance to oxidative loads, is a paradigmatic example of how cell homeostasis works. The 1CC, being the pathway for adding the carbon units to molecules, ensures growth and differentiation of the cells and has a kind of metabolic priority. Within the pathways fed by the 1CC, DNA methylation has a further priority. The ability to defend the cell against oxidations is thereafter relevant only for cells that are duly growing with a positive balance of activated methyl groups (SAMe) to switch on GSH synthesis, otherwise the cell will be addressed to abortive apoptosis. The final effect is that the antioxidant defences will be active and efficient only in cells that are metabolically healthy.

### Issues with oral antioxidants

There is good consensus that oxidative damage to male gametes represents a relevant clinical issue [33], which opens the idea that supplementation with oral antioxidants might improve gamete quality and the chance to achieve a pregnancy. Accordingly, several papers aimed to test this hypothesis published during the last 15 years consistently showed some positive effects of oral antioxidants on male gametes, either on sperm motility or on sperm morphology. Nevertheless, the latest Cochrane review on the antioxidant treatment of male infertility [34] concluded for low quality evidence that antioxidant supplementation in subfertile males may improve live birth rates and clinical pregnancy rates for couples attending fertility clinics. The same considerations apply to female infertility where clinical data in support are even weaker [35]. This could be probably ascribed to heterogeneity/weakness in studies design. In addition, speaking of male treatments, the ability of the oocyte of concern to repair damages to sperm DNA [4] may act as a confounding factor when assessing the treatment effect on the male side. However, metabolic issues may also apply.

The small effect on pregnancy rates is in part explained by the aggressiveness of oral antioxidants, forcing the cellular environment to the redox state, in front of the delicate metabolism of gametes that necessitate a perfect oxy-redox balance. Strong antioxidant cocktails may cause an excessive removal of oxygen free radicals essential for the regulation of several sperm functions: this may negatively affect capacitation and the acrosome reaction [36] and may induce a “reductive stress” as a rebound effect [37]. This was clinically confirmed by the worsening of the sperm nuclear condensation and padlocking of the protamines, a very negative outcome, that proportionally mirrored the improvements of the DNA fragmentation when strong antioxidants (Vit. C + Vit E + beta-carotene + zinc + selenium) were administered to infertile men [38].

It is anticipated that the reductive stress will interfere with the process of protamination and DNA synthesis. Both processes imply the resolution of the so-called supercoiling of DNA, which happens by extensive cut and paste work by the enzymes topoisomerase 2B (TOP2B) and poly-ADP-ribose-polymerase (PARP) [39]. TOP2B generates doublestrand breaks to allow distension of the double helix. Thereafter PARP adds to the break a chain of poly-ADP-ribose (PAR) that causes detachment of TOP2B and allows the process of recatenation. PARP consumes huge amounts of oxidised NAD (NAD+) as the ribose source for the synthesis of PAR: as DNA replication starts there is a sharp fall in cell NAD+ content and activation of ATP hydrolysis to feed NAD+ availability [40]. Any reductive stress will cause imbalance of NAD toward its reduced form (NADH) [41] resulting in shortage of NAD+, in metabolic inhibition of PARP and in accumulation of unrepaired double strand breaks for imbalanced TOP2B activity.

Another problem is that, whatever the composition of antioxidant cocktails, it will have to contain a finite number of substances. Each of these substances will influence a finite number of oxy-redox reactions that, according to the dose of antioxidants administered, will be possibly imbalanced toward the reductive stress. Meantime, all the other oxy-redox reactions will remain unaffected. The final outcome is failure in equilibrating the intracellular environment and a possible reductive stress in selected pathways. This approach may be clinically advantageous any time the objective is the correction of a specific, well-known pathway but it is not suitable if the aim is to achieve a perfect oxy-redox balance and efficient DNA replication and protamination as required by gametogenesis.

In summary, as the harmful effect of oxidative stress on gametes health is unquestionable and assumed the need to counteract the oxidative aggression in the sake of improved gametes quality, alternatives to the straight oral antioxidants administration are worthy to be explored.

### How to feed and stimulate the antioxidant system

Once understood, the role of the 1CC in the activation of the physiologic antioxidant responses, it can be postulated that a robust support to the 1CC could result in improved DNA methylation, sustaining gametes maturation, and activation of the endogenous antioxidant system, configuring a physiologic and self-regulated antioxidant activity devoid of any risk of “reductive stress”. The 1CC is strictly dependant on dietary sources of methyl donors and of other essential substances, absolute or relative shortages may occur. In addition, a heavier endogenous (e.g. inflammation) or exogenous (e.g. pollutants) load of oxidative aggression may be responsible for a relative shortage also in individuals otherwise following a relatively healthy lifestyle.

The list of dietary substances useful to sustain the 1CC is quite short and simple. Folates are to be included as the source for methyl groups for Hcy re-methylation. Quantities of riboflavin (vit. B2) and niacin (vit. B3) will support the conversion of folic acid to its active reduced form by the enzyme Methyl-Tetra-Hydro-Folate-Reductase (MTHFR). Vitamin B12 is then necessary to pass the methyl group from folates to Hcy. Finally, zinc, that has no body reservoirs and is strictly dependant on daily feeding [42], is also necessary because the main enzymes involved (MTHFR, MTRR, CBS) work by means of zinc fingers. It is to be noted that only chelated zinc is actually bio available and that most of the commercial supplements contain zinc oxide that provides a negligible support [43]. The above substances in due amounts have the potential to saturate the 1CC and to generate an excess of activated methyl groups (SAMe) for the activation of CBS and for an efficient GSH output anytime it is needed. GSH synthesis can be further supported providing its necessary co-factor pyridoxine (vit. B6) and, again zinc. In addition, a cysteine donor (e.g. N-acetyl-cysteine or l-cystine) has the potential to feed GSH synthesis downstream to CBS and to allow the enlargement of the GSH-GSSG pool if it is needed.

### Clinical results

A nutritional supplement containing all the substances necessary to support the 1CC (vit. B2, B3, B9, B12 and chelated zinc) and the GSH production (cysteine donor, vit. B6 and zinc) was formulated and addressed to clinical trials. The supplement also contained small amounts of Vit. E (12 mg) as a stabilizer (to prevent premature oxidation of the cysteine donor) and betalain and quercetin of vegetal origin to protect membrane lipids (Condensyl^TM^). Indeed betalaines have the property to stratify on plasma membranes where they act as suicide oxidation substrates to prevent the trigger of the lipoperoxidative cascade so to further aid sperm motility. This nutritional intervention was tested in male [44–46] and female [46] partners in couples with previous IVF/ICSI failures willing to undergo another ART attempt. These studies included couples with at least 2 previous ART failures and with a male partner showing sperm DNA Fragmentation Index (DFI—Measure of oxidative molecular damage, as assessed by TUNEL) and/or a Sperm nuclear Decondensation Index (SDI—Measure of integrity of chromatin tertiary structure, as assessed by aniline blue staining) > 20 % irrespective of the other sperm parameters. A 4-month nutritional support was prescribed.

The very first study was intended to confirm that: (i) The above formula does indeed exert antioxidant modulation in clinically significant amounts, and; (ii) The antioxidant gain does not generate any reductive stress as previously seen with oral antioxidants [38]. A total of 84 patients fulfilled the entry criteria of this non comparative study [44], in 28 cases (33 %) there was an associated female factor. For the first time ever, both sperm indexes significantly improved (DFI from 29.7 to 23.1 %, *p* < 0.001; SDI from 40.1 to 36.3 %, *p* < 0.001) confirming that the new support strategy is effective in counteracting oxidative damage without negatively influencing (reductive stress) the nuclear decondensation. The study also recorded the pregnancy outcomes and, given the inclusion criteria, the results were encouraging. Eighteen couples (21 %) experienced a spontaneous pregnancy during the treatment, all of them ending with a live birth. The remaining 66 couples underwent a new ART attempt further resulting in 22 clinical pregnancies and in 15 live births. The overall clinical pregnancy rate (CPR) and live birth rate (LBR) were 47.6 and 39.3 %, respectively.

The pregnancies seemed to be strongly related to the improvement of the sperm decondensation index (Table 1) but the small sample size did not allow the calculation of a SDI cut-off value predictive of a successful pregnancy. The higher predictive value of the SDI compared to the DFI was expected. A well-compacted chromatin will protect the chromosomes from the oxidative burst of the acrosome reaction at time of fertilization [47]; thus, it has a direct link with the ability to fecundate by natural routes, which accounts for the spontaneous pregnancies, and with the ability of the sperm to activate the embryo. On the other side, the oxidative damage to the genes, as reported by extensive DNA fragmentation, will have a strong impact at time of expressing such genes, i.e. on embryo viability, leading in case to pregnancy loss that could not be detected with the study model of concern.

**Table 1:** DFI and SDI response to Condensyl according to clinical pregnancies, mean values, Mann-Whitney test [44]

Groups	n (%)	DFI				SDI			
		Pre	Post	Δ %	p	Pre	Post	Δ %	*p*
Any pregnancy									
YES	40 (47.6)	29.4 %	20.1 %	−9.3 %	0.168	40.6 %	29.3 %	−11.3 %	*0.000*
NO	44 (52.4)	30.1 %	25.9 %	−4.2 %		39.6 %	42.6 %	3.0 %	
Spontaneous pregnancy									
YES	18 (21.5)	23.2 %	18.4 %	−4.8 %	0.571	44.8 %	29.8 %	−15.0 %	*0.000*
NO	66 (78.5)	31.5 %	24.4 %	−7.2 %		38.8 %	38.0 %	−0.7 %	
ART pregnancy									
YES	22 (33)	34.4 %	21.4 %	−13.0 %	*0.046*	37.2 %	29.0 %	−8.2 %	*0.001*
NO	44 (67)	30.1 %	25.9 %	−4.2 %		39.6 %	42.6 %	3.0 %	

The intervention was then tested in comparison with a non-treatment group using the same supplement [45] or a similar one including also small amounts of natural direct antioxidants (Procrelia^TM^) [46]. The second comparative study [45] included a female treatment arm: supplements were given to couples with previous ART failures due to known female factor. It is to be noted that in both the comparative studies the controls were patients not willing to take antioxidants, thus the studies were not truly randomized. In male treatments [45, 46], again, both the DFI and the SDI significantly decreased whereas they did not change in their controls. It is noteworthy that, differently from sperm parameters that show intra-individual variability, these indexes of sperm damage are rather stable over time without interventions. The pregnancy rates were significantly higher in the treatment groups in all study arms compared to untreated arms with a high rate of pregnancies occurring spontaneously. Surprisingly, the pregnancy gain from the female treatment [46] was at least as high as the one recorded with male treatments, suggesting that the impact of oxidative damage on female infertility may be similar if not even larger. The pregnancy outcomes of the male partners and their controls [44, 45] and of the female partners and their controls [46] are summarized in Table 2.

**Table 2:** Comparative studies [45, 46]: pregnancies and deliveries, mean values, Chi square test

	*N* of pts	Pregnancies, n (%)		Deliveries	*p*
		All	Spontaneous		
Only men treated, female partner “normal” [45]					
Treated	69	35 (50.7 %)	10 (29 %)	29 (42 %)	*0.003*
Controls	83	23 (27.4 %)	0 (0 %)	18 (21 %)	
Only men treated, female partner “normal” [46]					
Treated	95	49 (56 %)	8 (16 %)	45 (47 %)	*0.001*
Controls	84	23 (27 %)	0 (0 %)	18 (21 %)	
Only women treated, male partner “normal” [46]					
Treated	100	45 (45 %)	30 (67 %)	40 (40 %)	*0.0001*
Controls	73	10 (14 %)	0 (0 %)	8 (10.9 %)	

Taken together, these data strongly support the idea that a tailored nutritional intervention in support of the 1CC and of GSH synthesis may have a fundamental impact on the reproductive prognosis of couples resistant to ART cycles and, in general, in infertile couples. The high rate of spontaneous pregnancies, not recorded in control groups, may in part result from a selection bias: repeated ART failures may have selected couples with a main metabolic problem. As a matter of fact, it has been demonstrated that morphologically normal spermatozoa with non-condensed chromatin may be found also in subjects with normal sperm parameters [48]. Therefore, some cases of idiopathic male infertility leading to unexpected ART failures may be due to high, but yet undemonstrated, SDI values due to metabolic reasons. Accordingly, adequate nutritional care offered since the first referral to both partners of couples with unexplained infertility might decrease the need for repeated ART cycles and may also help to increase the chances of IUI.

The strong relationship between the occurrence of a pregnancy and the improvement of sperm nuclear condensation in the male partner (Table 1) may represent further proof of the key role of chromatin structure in gametes’ reproductive competence. The positive outcomes obtained in women may suggest that a similar effect is also exerted on female gametes, although the lack of clinically suitable DNA damage tests does not allow final confirmation.

Based on the negative effects of direct antioxidants on sperm nuclear condensation [38], their indiscriminate use should be avoided. These treatments should be prescribed only to patients with confirmed damage under strict medical control. On the other side, nutritional support based on the modulation of the physiologic antioxidant system, e.g. Condensyl, did not exert any counter effect and, on the contrary, improved the sperm nuclear decondensation while significantly reducing DNA fragmentation. These treatments could hence be offered to a larger population before seeking pregnancy and without fixed limits of duration.

### Does MTHFR genotype affect the outcome?

Once the clinical opportunities arising from a tailored supplementation to the 1CC are understood, the next question is whether such supplementation would benefit the carriers of a deficient variant of the enzyme Methyl-Tetra-Hydro-Folate-Reductase (MTHFR). The enzyme variants are thermolabile, have a more rapid turn-over and exert reduced activity. The deficit of the MHTFR function according to the genotype is reported in Table 3 [49].

**Table 3:** Deficit of the MHTFR function according to the genotype. From van der Put et al. 1998 [49]

Genotype	677CC 2 normal 677C	677CT Heterozygous 677 T	677 TT Homozygous 677 T
1298AA 2 normal 1298A	100 %	66 %%	25 %
1298 AC Heterozygous 1298C	83 %	48 %	Not tested
1298CC Homozygous 1298C	61 %	Not tested	Not tested

Genetic variants of the MTHFR gene indeed have a high prevalence. The widely investigated C766T mutation has a variable prevalence according to ethnicity and geographical areas following a clear-cut north to south gradient with an incidence of the TT homozygotes in newborns ranging from <5 % in Northern Europe and Canada to >20 % in Southern Europe and Mexico [50]. However, also the A1298C mutation is common and sometime associated to the C766T mutation. According to a well-designed study [51], analysing the genotype of a cohort of consecutive newborns in Southern Italy, the C766T mutation was present in 79 out of 104 and in 23 of them it was homozygous. In addition, 55 out of 104 carried the A1298C mutation which was homozygous in 13 of them. A combined C766T and A1298C mutation was found in 28 % of the tested newborns whereas triple mutations were rare and a double homozygous state was not found, it is likely non-vital.

Recent metanalysis studies concluded for the MTHFR C677T [52, 53] and A1298C [54] mutations to be a risk factor for male infertility. The MTHFR genetic variants are also associated to female unexplained infertility [55] and have been reported to cause a reduced number of retrieved oocytes in women undergoing ART, which was corrected by increasing the dose of supplemented folic acid [56]. Nevertheless, carriers of MTHFR C677T variant have been shown to be resistant to standard folic acid supplementation requiring higher doses to achieve less pronounced blood Hcy reductions [57].

Thus, genetic variants may be a resistance factor to the treatment and a series of issues need to be clarified. Should we test the patients for MTHFR genotype? What do we do for homozygous subjects? Can we do better for heterozygous ones? Are there other genes involved?

### Beyond MTHFR variants

MTHFR is not the only enzyme of the 1CC occurring with a genetic polymorphism of functional consequences, rather the pathway is a concentrate of potential variants.

The enzyme methionine synthase (MTR) catalyzes the methylation of Hcy to generate methionine. In case of defective activity there is lack of Hcy re-cycling. In addition, it is the only enzyme that uses 5-methyltetrahydrofolate and deficient activity also results in the trapping of cellular folate as 5-methyltetrahydrofolate [58], which becomes unavailable for other folate-dependent reactions involved in purine and pyrimidine biosynthesis. The P1173L mutation, which results in replacement of proline by leucine at position 1173 of the amino acid sequence, causes megaloblastic anaemia and developmental delay by the age of year 2; however, a series of other mutations causing only a decreased activity of the enzyme due to shorter half-life have also been described [59]. Among these, the A2756G mutation, that is known to cause hyperhomocysteinemia, may circulate with high frequency: among an unselected groups of 125 Turkish children the AG heterozygous state had an incidence of 38 % and 5 % of them were GG homozygous [60].

The enzyme methionine synthase reductase (MTRR) is responsible of the reductive activation of MTR. Deficient MTRR activity causes hyperhomocysteinemia and all symptoms of B12 shortage even in presence of normal B12 levels. The defective MTRR variant A66G has been clinically linked with high Hcy and occurs with a frequency almost as high as MTHFR variants: according to a very large survey study in the USA almost half of the USA population was heterozygous for MTRR A66G with the highest prevalence in the non-Hispanic white ethnic group [61].

The enzyme betaine homocysteine methyltransferase (BHMT) is responsible of Hcy methylation using betaine (trimethylglycine) as the methyl donor. A long list of single nucleotide polymorphisms (SNPs) has been described, all of them implicated with the occurrence of reproductive, neoplastic and degenerative diseases, but always within variable associations with other SNPs of the same gene or of other genes of metabolic enzymes configuring a kind of genetic puzzle. In example, the G742A mutation was associated with the occurrence of any neural tube defect (NTD) in a USA population but only in the subgroup of mothers supplemented with folic acid whereas in non-supplemented mothers it had no influence [62], suggesting that a BHMT defect may be relevant only in conditions of folate shortage. In contrast, the same G742A mutation of BHMT, which occurred respectively in 17 and 7 % of control and case mothers in Canada, was shown to provide genetic protection against spina bifida [63]. Thus, very likely the functional effect of this variant, and possibly of others, depends on the variable associations with other genetic variants and with the feeding habits. Adding further complexity, the enzyme choline dehydrogenase (CHDH), responsible to provide the betaine substrate to BHMT and primarily involved in the 1CC, may commonly occur with the G233T mutation that was proven to cause motility, structure and energy deficits in sperms of transgenic mice [64]. As a matter of fact, the absolute prevalence of the BHMT and CHDH variants and of their association with other enzyme variants is not known and their phenotypic and functional outcome remains unpredictable.

As many as 150 clinically relevant mutations of CBS, the key enzyme for oxy-redox homeostasis, have been described [65]. In most of the cases, they are non-sense mutations and also multiple alternatively spliced transcript variants may occur. The homozygous state for several of these genes is responsible for homocystinuria, a disease leading to death within the age of 20–30 years. The most common of these mutations, a substitution of threonine for isoleucine at codon 278 (I278T), was found to occur in heterozygous form in 11.7 % of a control population [66]. It can lead to full homocystinuria or complete compensation according to the homo- or heterozygous state and to the combination with other genetic and protein splice variants but is usually associated with mild disease [67] and may account for many subclinical disturbances of the 1CC and of Hcy homeostasis. Many other single mutations leading to a defective, although non-lethal, function may occur alone or in association with others so that the genotype-function relationship of this enzyme remains difficult to predict.

**Fig. 4:**
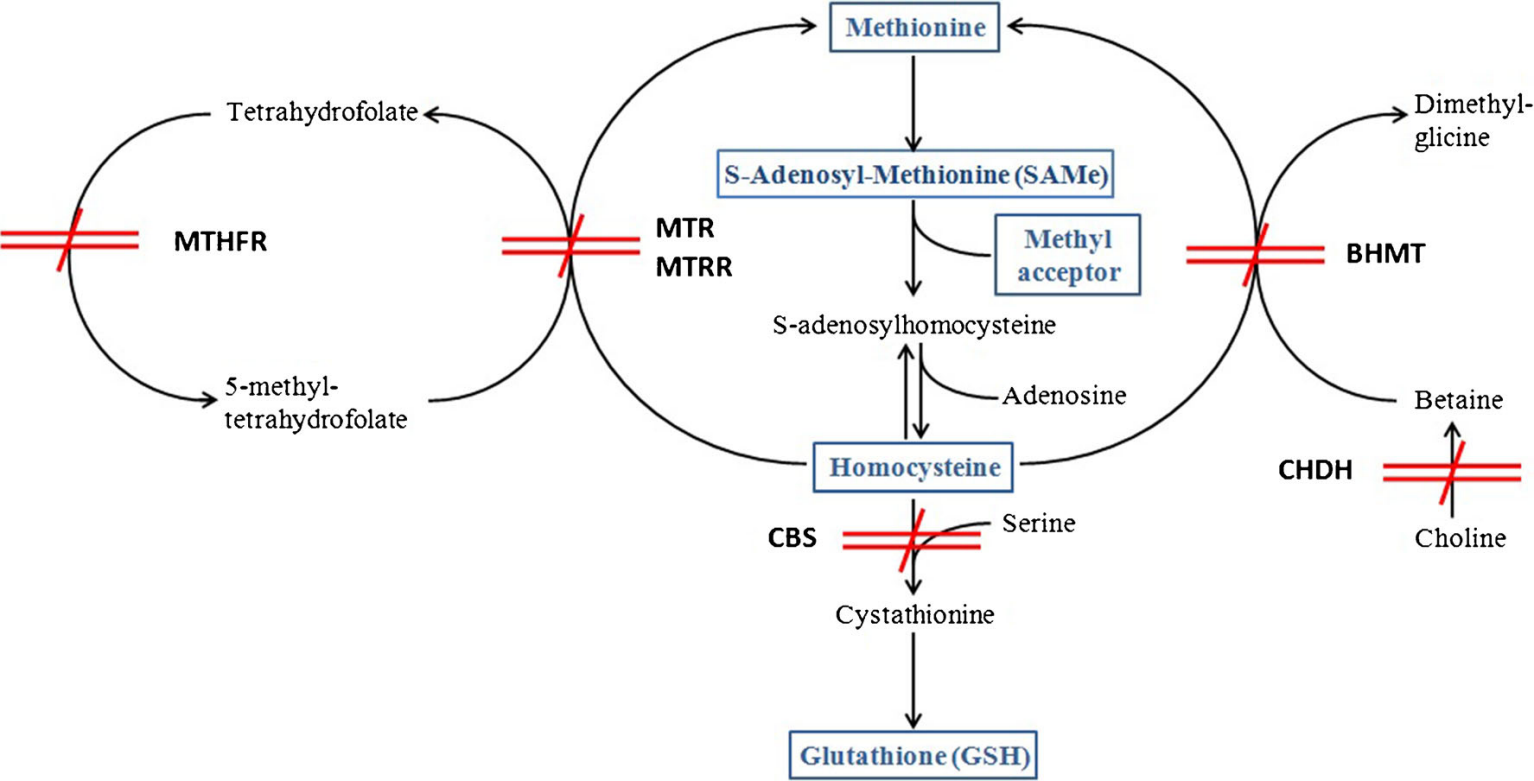
Schematic map of the possible genetic blockades to the 1CC and the GSH synthesis. All of the enzyme variants of concern have a high prevalence in the population and the chances for multiple defects are accordingly high. The final phenotype, i.e. the function of the carbon metabolism and of the antioxidant defenses, will depend on the variable combination of these genetic substrates with environmental factors including the diet and intercurrent diseases. *MTHFR* Methyl-Tetra-Hydro-Folate-Reductase, *MTR* Methionine Synthase, *MTRR* Methionine Synthase Reductase, *BHMT* Betaine Homocysteine Methyl Transferase, *CBS* Cistathionine Beta Synthase, *CHDH* Choline Dehydrogenase

A schematic map of the possible genetic blockades to the 1CC and the GSH synthesis is depicted in Fig. 4. The picture does of course include only the primarily involved enzymes, the list of secondarily involved enzymes and of their variants is almost unlimited. Further complexity is coming from the possible epigenetic regulation of the level of expression of these enzymes, from their mRNA regulation by means of non-coding RNAs and from the mostly unknown splicing variants of the encoded proteins.

This complexity explains why a carrier of a heterozygous MTHFR defective gene may be either well compensated or seriously metabolically impaired depending on the status of the other enzymes. Accordingly, the practice to extensively test the MTHFR genetic status in subfertile men and women is a kind of non-sense. It is clear that any individual risk assessment would require testing a variety of SNPs from a variety of possibly involved genes, and we do not even know how many genes are potentially involved neither we are sure of which are the SNPs relevant to functional defects. Even less is known about the phenotype and function level resulting from their highly variable associations. In our opinion, it is a lot simpler to test blood Hcy in both partners of all subfertile couples deserving the MTHFR gene analysis to those resulting with frankly elevated Hcy levels, i.e. above 30 μmol/L. Those homozygous for low activity variants will benefit from the diagnostic information that unmasks a series of risk factors, e.g. thromboembolic risk and neurodegeneration. For all the others, any wild type or variant heterozygous state will not mark any difference, at least as long as we will remain unable to interpret a so complex picture.

### Lessons from the gene-environment interaction

MTHFR variants are clearly linked to infertility and reproductive pathologies as well as to many life-shortening metabolic, degenerative and neoplastic diseases; thus, they should disappear within a few generations. Conversely, the variants can be found in a large part of every populations. How is this possible?

The answer from the geneticists is clear and final: This is the best possible demonstration of the gene-environment interaction. The prevalence of defective MTHFR variants clearly increases following a North to South gradient, i.e. the higher the consumption of a Mediterranean type diet, the higher the frequency of the defective MTHFR enzyme [50]. Besides the permissive action of the diet, the high prevalence of MTHFR variants also implies possible positive selection mechanisms [68], i.e. the variants may carry advantages. Indeed, cancer is known to be in some cases folate-dependent and to benefit from an efficient 1CC. Both the main MTHFR variants have been indicated as a possible resistance factors to colon cancer [69] and to metastases of myeloid leukaemia [70], just to quote some examples. In addition, the proliferation of the plasmodium of malaria is far less efficient in presence of a defective MTHFR and this may explain the high prevalence of these genes in the Mediterranean area [71]. In conclusion, a subject carrying a MTHFR variant that is well compensated by the diet and by the pattern of associated genetic variants of the other metabolic enzymes may have a selective advantage compared to what is considered a “normal” genotype. In example, the prevalence of MTHFR A1298C, MTR A2756G and of MTRR A66G, all of them considered defective variants marking a lower methylation capacity, was significantly higher in 77 elite athletes as compared to their sedentary controls [72]. A possible speculation is that a lower rate of homocysteine re-methylation to methionine may funnel homocysteine toward transsfulfurations and GSH production providing better balance to the increased ROS production due to a high energy balance in intensively trained subjects.

For historical and cultural reasons, MTHFR is far better investigated but it is very likely that similar considerations may come true also for the other enzymes of concern. Thus, we should refer to all these functional variants of the metabolic enzymes as population variants, i.e. a genetic diversity creating strength in the concerned species, including humans. Apart a few exceptions, e.g. MTHFR C677T homozygosity and homocystinuria, none of these mutations constitutes a pathologic trait per se.

**Fig. 5:**
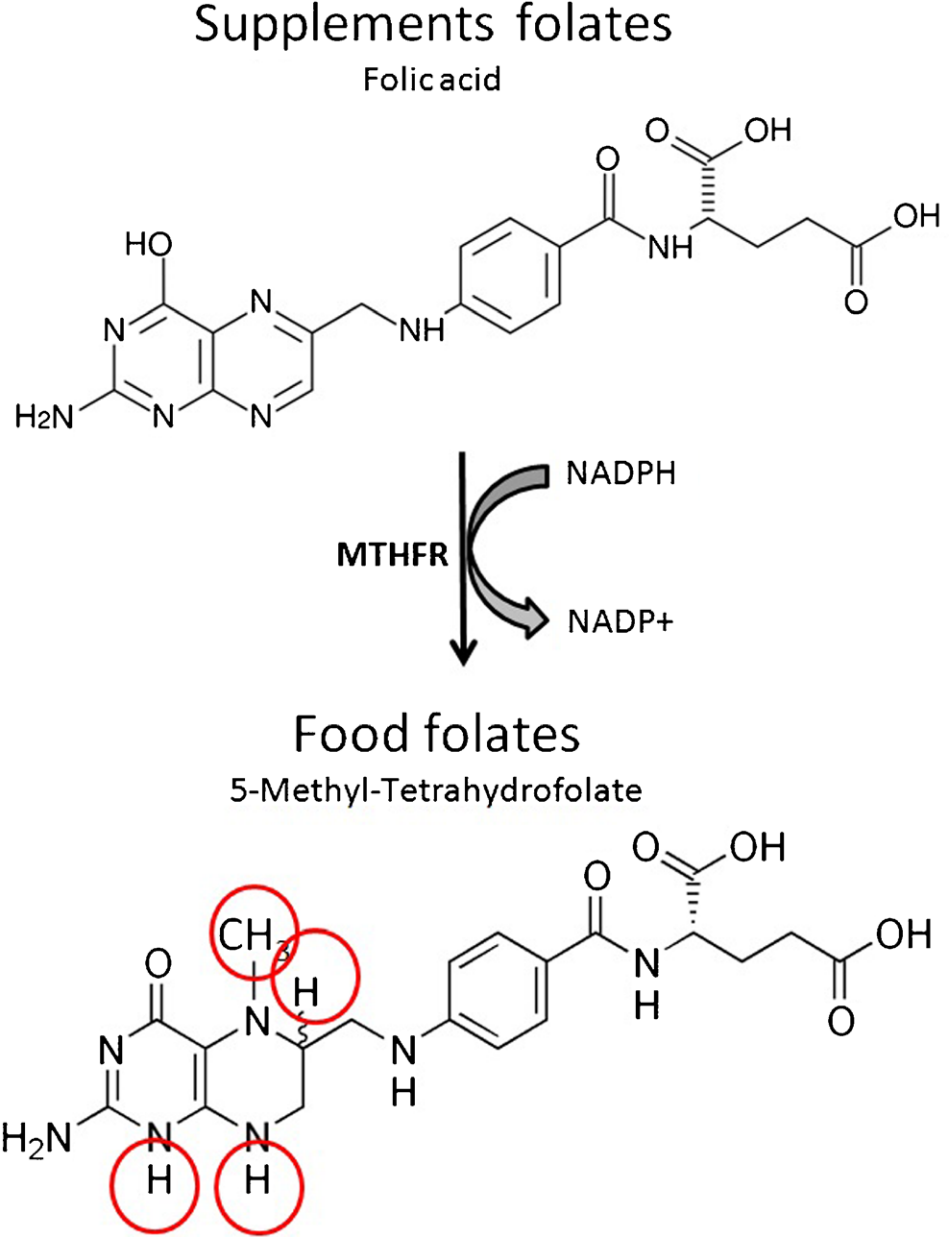
Food folates compared to supplement folates. The folates found in food consist of a mixture of reduced folates, mainly 5-Methyl-Tetrahydrofolate as shown in the figure. They also come with a polyglutamate tail (not shown), further increasing the solubility, whose length varies according to the type of food. Dietary supplements and fortified foods usually contain the synthetic form folic acid that is better stable, cheaper and easier to manufacture. Folic acid requires enzymatic reduction by the enzyme MTHFR (see *red circles*) to become soluble and bioavailable to cell metabolism. Subjects with a deficient MTHFR activity may not be able to process loads of the synthetic form. It is also to be noted that the reduction of folic acid from supplements consumes proportional amounts of NADPH and may be further hampered by any oxidative stress causing imbalance of NADP toward the oxidised form NADP+

The next point is to understand why Mediterranean diet compensates a lower MTHFR activity. The folates contained in many natural foods including meats, in all the fortified foods and in the vast majority of the dietary supplements are in the form of folic or folinic acid that necessitates MTHFR activity to be biologically effective. Lettuce, beans, tomatoes, broccoli and most of the vegetables typical of the Mediterranean diet contain the soluble and active form of folates methyl-tetrahydrofolate [73], i.e. the reduced form that does not require the activation by MTHFR (Fig. 5). Thus, a high dietary intake of these vegetables feeds the 1CC and the activation of antioxidant defences irrespectively of MTHFR activity. Once compensated by the diet the defective MTHFR becomes an advantage and its prevalence increases. Conversely, the Mediterranean population should be warned that the contemporary trend to quit the traditional feeding habits may cause the loss of these metabolic advantages and may generate reproductive issues.

On the other side meats are rich in the as well necessary B12 and zinc. Indeed, in spite of a potentially high intake of methyl-tetrahydrofolate, vegans develop fast onset high blood homocysteine. Thus, the more the diet is varied and comprehensive of all type of foods, the higher are the chances to achieve a balanced support of nutrients independently of the individual genetic inheritance of enzymes alleles and to enjoy well efficient 1CC and antioxidant defences.

### Issues with folic acid supplementation: un-metabolized folic acid (UMFA)

Healthy feeding habits and lifestyles are the basic requisite for a good health and for efficient reproductive function, but in many instances it may be not enough. The modern lifestyle makes the adherence to a well varied diet difficult and engaging. In addition, the toxic load from the environment is increasing and may peak in subjects with known (e.g. occupational) or unknown exposures. Needless to say, subjects homozygous for any defective variants of the enzymes or carrying an imbalanced pattern of heterozygosis for several of the enzymes are further exposed to relative dietary shortages.

This is favoring an increasing haphazard use of dietary supplements aimed to compensate known or supposed defects, which causes several concerns about a possible over exposure to folic acid. Indeed every manufacturer of dietary supplements is including some folic acid by default. Thus, people consuming more than one supplement, which is very common [74], accumulate folates and can easily go far above 1 milligramme per day. In addition, more and more food fortification public programs are in place and many licenses for branded foods fortified with folates have been released. The population of Western countries is nowadays overloaded with folic acid.

Reduced folates, namely 5-methyltetrahydrofolate, are the soluble/circulating and bioavailable form of this nutrient (Fig. 5). The occurrence into the circulation of the non-reduced forms, usually termed UMFA (UnMetabolized Folic Acid) is the result of lack of activation and may create metabolic imbalances. A recent survey conducted by the National Center for Health Statistics (NCHS) in the USA found circulating UMFA in nearly 100 % of the tested population, independent of their age or ethnic group, and also in non users of food supplements [75].

UMFAs are completely ineffective in feeding the 1CC and, once accumulated, they are predicted to be diverted to pyridine synthesis with the potential to overfeed growing cancers. Although there is no evidence that an excess of UMFA would directly cause any cancers, the risk to accelerate growth and metastases of already existing cancers is real [76]. This is predicted by mathematical models [77] and already shown in the clinical setting for colon adenomas [78] and breast cancer [79]. Moreover, UMFA may interfere with immune functions and have been shown to cause a decrease in natural killer activity [80]. As a matter of fact, even if folic acid is inexpensive and easy to manufacture, food fortification programs have been questioned both in Europe [81] and in the USA [75].

Subjects carrying any defective MTHFR variant, being unable to metabolize/activate folic acid, are highly exposed to circulating UMFA and to their clinical consequences. Accordingly, high folic acid intake should be avoided in these subjects. In reality, we are doing just the opposite: it is common practice to supplement the carriers of a homozygous MTHFR variant with huge doses of folic acid to decrease their blood Hcy [82]. The mega dose of folic acid, usually 5 mg corresponding to 25-fold the daily requirements, creates a huge excess of substrate and is able to normalise the Hcy levels in many subjects. Although this supplement is cheap and reimbursed by the national health services in several countries, this overuse in our opinion constitutes a metabolic nonsense. We are overloading with folic acid exactly those subjects that cannot metabolise it, i.e. those subjects where the negative outcomes of the UMFA syndrome are expected to peak. As long as we are unclear about the medium and long term consequences of UMFA exposure, this should be avoided and alternative remedies, i.e. support extended to the alternative pathways, should be pursued.

### Future directions

In spite of the above complexity, better effective interventions may be easy to implement. Even if a personalised risk assessment is not possible to date, it is already possible to integrate the diet so that all of the main potential defects get compensated in the best possible way whatever the genetic substrate.

The MHTFR variant has no influence on the performance of the 1CC if the diet is rich in reduced folates (raw vegetables), i.e. methyltetrahydrofolate, which is the soluble, active form and that does not require MTHFR activation [83]. The defective variants of MTR and MTRR can be compensated by consumption of vitamin B12 in its active form, methylcobalamin (milk, meats, oilly fish), that will support Hcy re-cycling from methyltetrahydrofolate independently of those enzymes [84]. The possible BHMT/CHDH activity deficiency can be compensated by an excess of substrate, i.e. consuming larger amounts of betaine and choline (cereals). Finally, CBS activity defects can be mitigated if the diet is rich in cysteine donors (sulphurated proteins from wheat, eggs, soy) that can feed GSH de-novo biosynthesis downstream to CBS independently of its activation. Such a varied dietary regimen, besides being of paramount importance for the carriers of any of the concerned enzymatic defects, would be just better than any imbalanced regimen also for those with a wild-type genetic substrate and should be therefore followed by everybody independently of any genetic testing.

The above wide range support of micronutrients can be achieved with a better attention to the diet and is also suitable for the formulation of supplements (see Table 4) [73, 85–90]. Accordingly, a dietary supplement containing the allowed amounts of the above substances, of course together with B2, B3, B6 and chelated zinc (Impryl) was formulated in tablet form and addressed to clinical testing as a support to female reproduction. As expected, this treatment is already showing the ability to normalise Hcy levels in carriers of the TT phenotype of MTHFR, including those that were partially or fully resistant to huge doses of folic acid. In addition, while clinical studies are ongoing, the treatment of open label case series is already providing interesting information, indeed this is also a tool to check the relevance of oxidative stress and/or enzyme variants in specific clinical conditions. We are recording a complete and fast regression of all the signs and symptoms from PCOS in most of the treated ladies, likely due to a decrease of the insulin resistance that is triggered by oxidative stress [91]. This is a strong argument in favor of a major role of oxidative damage in the disease. The support also favored the increase of the Anti-Mullerian Hormone (AMH) levels and the decrease of circulating FSH in ladies of advanced reproductive age, which seems to imply some oxidative mechanisms affecting the hypothalamus-pituitary axis in the onset of ovarian failure. Indeed, methylfolate is the only folate form permeating the blood-brain barrier and also methylcobalamin passes with far higher efficacy compared to standard B12. Thus, the latter outcomes may be directly related to the modulation of brain metabolism leading to the concept that some reproductive dysfunctions may depend on oxidative stress within the brain.

**Table 4:** Supplement (Impryl) ingredients and the respective food sources [73, 85–89]

Supplement ingredients		Food sources
Ingredient	Daily dose	
Betaine	200 mg	Cereals (wheat bran/germ/bread), shrimps, spinach [85]
L-cystine	200 mg	Cysteine: Proteins (sulphurated) from wheat, eggs, soy, meat [86] and whey/casein [87]
Niacin (vit. B3)	16 mg	Milk, eggs, rice, fish, lean meats, legumes [88]
Zinc	10 mg	Seafood (crustaceans, seashells), red meat (lamb), spinach [89]
Pyridoxine (vit. B6)	1.4 mg	Poultry, fish, organ meats, potatoes, fruit [89]
Riboflavin (vit. B2)	1.4 mg	Eggs, organ meats, lean meats, milk, Green vegetables [89]
Methylfolate (vit. B9)	400 μg	Green vegetables (e.g. spinach, lettuce, broccoli, endive and radicchio) [73], tomatoes [90]
Methylcobalamin (vit. B12)	2.5 μg	Any cobalamin: Fish (seashell, oily fish), meat (beef liver), poultry, eggs, milk, cheese [89]. Actual content in methylcobalamin is not known.

Supplement ingredient amounts are in line with the Nutrient Reference Values of the European Food Safety Agency, where applicable. It is to be noted that to provide the whole amount of micronutrients contained in the supplement more than one standard serving of the suggested foods may be needed. However, a single food, e.g. eggs, may contain several of the concerned micronutrients

Prospective clinical trials are needed to assess the actual benefits and limits of this approach, meanwhile, due attention to the diet so as to include as many as possible different food sources is highly recommended.

### Conclusions: Dear Practitioner…

In summary, there is a complex interaction between the environment, genes and our feeding habits. The individual outcome depends on a variety of genetic, epigenetic and behavioral factors. Clinically relevant damage is commonly occurring, mainly dependent on perturbations of the 1CC and has the deregulation of DNA re-arrangements among the most deleterious effects. The diet, in case associated to tailored supplementations, has the potential to adjust most of the imbalances in most of the subjects.

Full understanding and proper management of all this aspects may have a fundamental impact on the reproductive prognosis as well as on global health. Pending further understanding of all the involved mechanisms, there are already good indications to be implemented in clinical practice:

- Oxidative damage is clinically important because it has the potential to hamper gamete reproductive competence and to increase the aneuploidy rate;
- Oral antioxidants are powerful tools with strong pharmaceutical effects but misuse occurs easily; their chances to achieve a metabolic balance are negligible;
- The key to achieve an oxy-redox balance is the support to cellular homeostatic mechanisms: Full availability of micronutrients necessary to the 1CC favors the synthesis of GSH and the function of the endogenous antioxidant cascade resulting in measurable clinical gains;
- High attention should be given to the damage to the chromatin structure and to the epigenetic regulation of the gametes both in the diagnostic phase and in monitoring the effects of any interventions;
- The individual genetic status has a primary impact on the susceptibility to the damage, but risk assessment of the individual remains difficult to define. The screening of Hcy levels can be of help in selecting patients that benefit from a detailed genetic investigation;
- Although cheap and easy, folic acid is just a pre-vitamin and excessive consumption may create health problems. Food fortification programs should be further re-considered. The prescription of huge doses to subjects that do not metabolise it, e.g. MTHFR variant homozygous, is a questionable idea;
- Only a balanced and very varied diet has the potential to fulfill the needs in all subjects, once achieved dietary supplements can be of further help. Whatever stressed diet, including vegetarian, hyper-proteic and the like, has the potential to create pathologic imbalances;
- The activated forms of micronutrients are of greater help and their requirements can be fulfilled with tailored dietary regimens. They can also be provided by dietary supplements;
- Due attention to the metabolic issues and supportive interventions since the first referral have the potential to reduce the time to pregnancy of infertile couples and to increase the final cumulative success rate.

## Glossary

1 Carbon Cycle (1CC): Also called transmethylation pathway, is a main pathway for non-photosynthetic organisms to add the carbon unit to molecules, it is essential for cell growth and differentiation, including DNA methylation. Upregulates transsulfurations.
Betaine Homocysteine Methyl Transferase (BHMT): Zinc metallo-enzyme that catalyzes the transfer of a methyl group from betaine (trimethylglycine) to homocysteine to form dimethylglycine and methionine. Provides folate-independent Hcy re-methylation.
Choline Dehydrogenase (CHDH): Oxidoreductases oxidising choline to betaine aldehyde, which is ultimately transformed to betaine by aldehyde dehydrogenase. Feeds the endogenous betaine availability for the BHMT reaction.
Cystathionine Beta Synthase (CBS): Redox-regulated enzyme having vit. B6 as essential co-factor and allosterically up-regulated by SAMe. It forms cystathionine from Hcy and serine thereby opening the transsulphuration pathway leading to GSH synthesis.
Glutathione (GSH): It is a tripeptide formed by cysteine, glycine and glutamate. Thanks to the -SH group from cysteine it is able to act as electron donor forming glutathione disulfide (GSSG). GSH de-novo biosynthesis occurs within the transsulfuration pathway.
Homocysteine (Hcy): It is a non-proteogenic aminoacid similar to cysteine and formed by de-methylation of methionine. Within the 1CC it is re-methylated to methionine by methyl groups donated by folates or betaine. It is also the substrate of CBS for the start of the transsulfuratuion pathway. Hcy accumulation is toxic to cells.
Methionine synthase (MTR): It is responsible for the remethylation of homocysteine from folates and uses vit. B12 as essential co-factor. After a reductive activation of the enzyme-bound cobalamin to methylcobalamin by MTRR, MTR passes the methyl group from methylfolate to Hcy forming methionine.
Methionine synthase reductase (MTRR): It is the enzyme responsible for the activation of MTR. It operates a reductive methylation of the oxidised form of the MTR-cobalamin complex using SAMe as methyl donor and NADPH as the electron donor.
Methyl-Tetra-Hydro-Folate-Reductase (MTHFR): Reductase containing a bound flavin cofactor (from riboflavine) and using NAD(P)H as the electron donor. MTHFR irreversibly reduces 5,10-methylenetetrahydrofolate to 5-methyltetrahydrofolate to feed the MTR-dependent re-methylation of Hcy (rate-limiting enzyme).
Nicotinamide adenine dinucleotide (NAD): Di-nucleotide acting as electron donor in oxy-redox reactions. Also occurring in its phosphate form (NADP) to serve specific enzymes. The equilibrium between its reduced (NADH/NADPH) and oxydised (NAD+/NADP+) form influences the direction of oxy-redox reactions.
S-Adenosyl-Methionine (SAMe): Ubiquitous substrate involved in in anabolic methyl group transfers and transsulfurations. It is formed within the 1CC by activating adenylation of methionine and, after releasing the methyl group to acceptors, forms S-adenosyl-homocysteine and thereafter Hcy. It occurs in any cells with a massive production and consumption in the liver.
Transsulfuration pathway (reverse): Pathway responsible for the passage of the -SH group from Hcy to cysteine. The opposite passage (forward) occurs only in bacteria. It is a relevant source of -SH groups for GSH synthesis and also contributes to the de-toxication of high Hcy. It upregulates the 1CC.
